# Evidence Summary of Pruritus Management in Patients With Atopic Dermatitis: Implications for Nursing Practice

**DOI:** 10.1111/jocd.71111

**Published:** 2026-08-31

**Authors:** Yuzhen Shen, Qian Shen, Manni Chen, Rufang Liu

**Affiliations:** ^1^ The Second Clinical Medical College of Guangzhou University of Chinese Medicine Guangzhou City China

**Keywords:** atopic dermatitis, evidence summary, nursing practice, patient education, pruritus, skin barrier

## Abstract

**Background:**

To synthesize the most current and relevant evidence regarding pruritus management in patients with atopic dermatitis (AD), and to identify implications for nursing assessment, education, monitoring, and follow‐up.

**Method:**

An extensive literature search was conducted across multiple databases, including the Cochrane Library, Embase, and PubMed in English, as well as Chinese databases such as WanFang Data and CNKI. The search encompassed publications from the inception of these databases up to December 2024.

**Results:**

A total of 26 publications were ultimately included in the analysis. These consisted of 14 randomized controlled trials, 3 systematic reviews, 2 cohort studies, 1 scoping review, 2 observational studies, 1 case report, 1 expert opinion, and 4 expert consensus documents. The findings were organized into physician‐directed therapies, nursing‐implemented or nursing‐supported interventions, and nursing‐specific practice implications; region‐specific or traditional therapies were presented separately because of their limited international generalizability. Key implications for nursing practice were identified in the areas of symptom assessment, skin barrier care, patient education, treatment adherence, safety monitoring, and follow‐up support. Nurses play an important supportive role in implementing physician‐directed therapies and non‐pharmacological interventions while also promoting adherence and long‐term disease management.

**Conclusion:**

Evidence‐based management of pruritus in atopic dermatitis requires integration of pharmacological and non‐pharmacological strategies. Nursing practice contributes substantially through symptom assessment, patient education, skin care support, adherence promotion, safety monitoring, and follow‐up, thereby helping improve pruritus control and quality of life.

## 1 Introduction

Atopic Dermatitis (AD) is a chronic inflammatory skin disorder characterized by recurrent eczematous lesions and severe pruritus [1]. The global prevalence of AD has risen significantly in recent decades, with epidemiological studies indicating that approximately one‐fifth of pediatric populations and 3%–5% of adults are affected by this condition. This condition often begins in early childhood, with around 60% of cases emerging within the first year of life and 80%–90% appearing before the age of five [2]. Although many children experience spontaneous resolution of symptoms prior to puberty, around 25% continue to endure the condition or experience relapses into adulthood [3]. The natural progression of AD exhibits distinct clinical manifestations at various life stages: in infancy, eczema predominantly affects the face, scalp, and the extensor surfaces of the limbs; as children mature, lesions often localize to flexural areas such as the elbows and knees; in adulthood, AD frequently presents as hand eczema or dermatitis localized to the head and neck [4].

Pruritus is the hallmark symptom of AD and represents one of the most distressing aspects for affected individuals. This itching not only disrupts daily activities, leading to limitations in routine functioning and social interactions, but it also significantly compromises sleep quality, potentially resulting in anxiety, depression, and a range of psychosocial issues [5]. Research indicates that worsening pruritus in AD is associated with progressively impaired quality of life, with more severe itching corresponding to greater declines in physical and psychosocial well‐being [6]. Moreover, patients with AD may encounter additional complications, including skin infections (notably caused by *Staphylococcus aureus* ), allergic rhinitis, and asthma, which further exacerbate their clinical burden [7, 8].

A hallmark feature of AD is epidermal barrier dysfunction, primarily driven by deficiencies in key structural components such as ceramides and filaggrin (FLG gene). This impairment results in elevated transepidermal water loss, xerosis, and heightened susceptibility to environmental allergens and microbial pathogens. This barrier defect allows allergen penetration, triggering immune responses and nerve activation, resulting in pruritus [9]. Itch transmission involves neural pathways, particularly transient receptor potential (TRP) channels like TRPV1 and TRPA1, which respond to inflammatory mediators, pH changes, and mechanical stimuli [10]. Neuropeptides, such as substance P, further amplify itch, creating a vicious cycle of “itch‐scratch‐inflammation.” Environmental factors like dust mites and pollen can worsen pruritus through immune and neural pathways, though AD‐related itch is mostly non‐histamine‐dependent. Managing pruritus in AD requires a multifaceted approach. Topical treatments include corticosteroids (TCS), calcineurin inhibitors (TCI), and JAK inhibitors, effective in flare‐ups but with risks like skin atrophy [9, 10]. Systemic therapies include antihistamines, immunosuppressants, and biologics, which modulate immune responses but require careful monitoring [11]. Non‐pharmacological interventions, such as phototherapy, wet wrap therapy, emollients, and psychological support, also play a crucial role in symptom relief [12]. An integrated approach combining these strategies is essential for effective pruritus control and improved patient outcomes [13].

Despite the array of available treatment options, the management of pruritus in AD continues to pose significant challenges. On one hand, a subset of patients exhibits suboptimal responses to existing therapies, complicating effective itch control; on the other hand, the chronic use of certain medications may lead to adverse effects, compromising patient adherence. Additionally, the heterogeneity of AD, characterized by varying etiologies and pathophysiological mechanisms among patients, further complicates treatment strategies. As frontline professionals involved in the management of AD, dermatology nurses contribute to multidimensional care through symptom assessment, skin care support, patient education, adherence promotion, safety monitoring, and follow‐up coordination. Although the initiation and adjustment of many pharmacological therapies remain physician‐directed, nursing expertise is essential for optimizing treatment implementation, identifying barriers to adherence, monitoring adverse effects, and supporting long‐term disease management.

Therefore, exploring and summarizing more effective approaches for managing pruritus is crucial for alleviating patient suffering, enhancing overall health outcomes, and improving quality of life. This review aims to summarize the current evidence on pruritus management in AD and to discuss its implications for nursing practice, with particular emphasis on symptom assessment, skin barrier care, patient education, adherence support, safety monitoring, and follow‐up.

## 2 Methods

### 2.1 Identification of Research Question

The clinical question was reformulated into an evidence‐based inquiry utilizing the PIPOST framework: (I) P (Population): patients diagnosed with AD; (II) I (Intervention): pharmacological and non‐pharmacological interventions related to pruritus management; (III) P (Professionals): multidisciplinary healthcare professionals involved in AD care, including nurses and physicians; (IV) O (Outcomes): improvement in pruritus severity, symptom burden, quality of life, and treatment adherence; (V) S (Settings): primary care settings, specialized ambulatory clinics, and acute care facilities; (VI) T (Type of Evidence): encompassing evidence‐based clinical protocols, Delphi‐derived consensus statements, position papers from professional societies, evidence‐based practice guidelines, comprehensive meta‐analytical studies, structured systematic evaluations, standardized care pathways, computerized clinical decision systems, peer‐reviewed technical white papers, and prospectively registered controlled clinical investigations.

### 2.2 Search Strategy

An extensive literature search was carried out utilizing a variety of databases, including the Cochrane Library, Embase, and PubMed in English, along with Chinese databases such as WanFang Data and CNKI. The search was limited to publications from the inception of these databases through December 2024. The search terms encompassed “atopic dermatitis,” “pruritus,” and related keywords and free text. For transparency and reproducibility, we pre‐specified detailed search strings; for example, the PubMed strategy was: (“atopic dermatitis”[Mesh] OR “atopic eczema”[tiab]) AND (pruritus[Mesh] OR itch[tiab]) AND (therapy[sh] OR treatment OR management), with analogous strategies adapted for Embase and the Cochrane Library. Chinese databases, including WanFang Data and CNKI, used combinations of subject headings and free‐text terms for “atopic dermatitis” and “pruritus”.

### 2.3 Inclusion Criteria

Eligible studies involved participants with a confirmed diagnosis of atopic dermatitis accompanied by pruritic symptoms [14]. The studies were required to focus specifically on management strategies for pruritus in atopic dermatitis and their effectiveness evaluations. Eligible study designs comprised high‐quality randomized controlled trials, cohort studies, case–control studies, systematic reviews, and meta‐analyses.

Exclusion criteria encompassed: study protocols, animal research, articles lacking accessible full texts, duplicate publications, and studies exhibiting significant methodological flaws (e.g., inadequate study design, small sample sizes, or questionable data integrity).

### 2.4 Study Selection

Literature screening was performed independently by two researchers trained in evidence‐based medicine. Any disagreements were resolved through detailed discussions or by consulting a third senior expert, thereby ensuring the objectivity and reliability of the selection process.

### 2.5 Evidence Extraction and Summary

The evidence‐based team members performed a thorough synthesis of the evidence guided by several principles: (I) when content was consistent, clearer and more understandable statements were prioritized; (II) complementary information from multiple sources was merged into a unified piece of evidence; (III) in instances of conflicting conclusions among different sources, higher regard was given to evidence based on quality, reliability, and the timeliness of authoritative publications. In addition, where available, we extracted information on minimal clinically important differences (MCIDs) for itch and quality‐of‐life scales and noted whether observed treatment effects met or exceeded these thresholds. When trials reported quantitative outcomes, we extracted absolute or relative effect sizes (e.g., mean differences in itch scores or risk ratios for response) together with their 95% confidence intervals to facilitate comparison across interventions. Where studies provided stratified results, subgroup data by baseline disease severity, age group or ethnicity were also recorded. Risk of bias for randomized controlled trials and cohort studies was assessed independently by two reviewers using the Cochrane risk‐of‐bias framework, with discrepancies resolved by discussion. We evaluated domains including random sequence generation, allocation concealment, blinding, incomplete outcome data and selective reporting; for non‐randomized and observational studies, we considered potential confounding, selection bias and outcome assessment. Many trials had at least one domain judged as high risk or some concerns, most commonly due to lack of blinding, small sample sizes or incomplete reporting. When synthesizing results, we therefore interpreted effect estimates cautiously and downgraded the certainty and recommendation strength for interventions supported mainly by small or high–risk‐of‐bias studies. The level of evidence was classified as follows: Level 1a for meta‐analyses or high‐quality systematic reviews of randomized trials; Level 1b for individual randomized controlled trials; Level 2a for systematic reviews of cohort studies; Level 2b for individual cohort studies; Level 3b for individual case–control or observational studies; Level 4 for case series or expert opinion; and Level 5 for expert consensus statements, guidelines, or position papers. Strength of recommendation was graded separately as A–D, with Grade A indicating strong recommendations supported by high‐quality, consistent, and directly applicable evidence; Grade B indicating moderate recommendations supported by generally consistent evidence of moderate quality; Grade C indicating weak recommendations based on limited, indirect, or inconsistent evidence; and Grade D indicating recommendations based primarily on expert consensus or very low‐certainty evidence. These definitions were applied consistently throughout the abstract, results tables, and figure notes, so that evidence level refers only to study design and evidence hierarchy, whereas recommendation strength refers only to the overall clinical recommendation. Detailed definitions of the level of evidence and strength of recommendation are provided in Table S2.

### 2.6 Statistical Methods

No primary statistical synthesis or meta‐analysis was performed in this evidence summary. For each included study that reported quantitative outcomes, we extracted effect measures as presented by the original authors. For continuous outcomes (e.g., pruritus intensity measured by Visual Analog Scale or Numerical Rating Scale), we extracted mean differences (MD) or standardized mean differences (SMD) with 95% confidence intervals (CIs) where available. For binary outcomes (e.g., proportion of patients achieving a predefined itch response), we extracted risk ratios (RR) or odds ratios (OR) with 95% CIs where reported. When a study provided only point estimates without CIs, we reported the estimate as available and noted the absence of precision measures. For studies that did not report quantitative between‐group comparisons, findings were described narratively.

## 3 Results

### 3.1 Literature Screening Process

**FIGURE 1:**
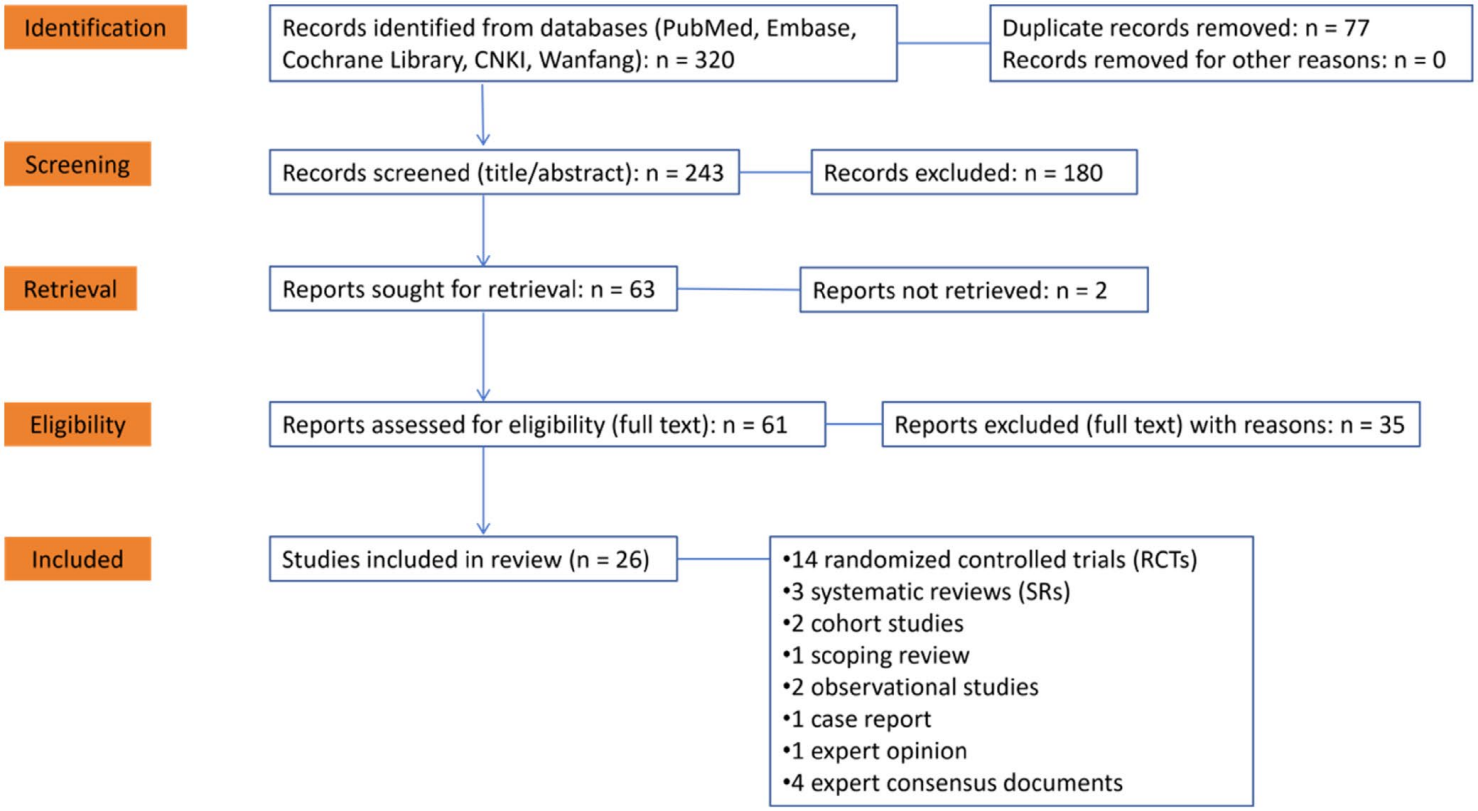
PRISMA flow diagram.

After the initial literature search and removal of duplicates, 243 publications remained. Following the exclusion of irrelevant articles based on titles, abstracts, and full texts, a total of 26 publications were ultimately included. The study selection process is depicted in a PRISMA flow diagram (Figure 1). Among these, the final selection comprised 14 randomized controlled trials, 3 systematic reviews, 2 cohort studies, 1 scoping review, 2 observational studies, 1 case report, 1 expert opinion, and 4 expert consensus documents. The essential information and levels of evidence for the included literature are summarized in Table 1, while recommendation strength for specific interventions is presented separately in Tables 2, 3, 4, 5. An overview of risk‐of‐bias assessments for randomized and cohort studies is provided in Table S1.

**TABLE 1:** Basic information of included studies.

First author	Publication year	Topic of literature	Study type	Evidence level
Wollenberg A	2020	Consensus on the diagnosis and treatment of atopic dermatitis	Guidelines/position paper	5
Sigurgeirsson B [15]	2015	Long‐term treatment with pimecrolimus	Randomized controlled trial (RCT)	1b
Silverberg JI [16]	2021	Tralokinumab combined with topical corticosteroids	Phase III double‐blind RCT	1b
Huang JT [17]	2009	Impact of *Staphylococcus aureus* colonization treatment on AD severity	Cohort study	2b
Matiz C [18]	2011	Risk of community‐acquired MRSA infection in AD patients	Observational cross‐sectional study	3b
Chiu LS [19]	2010	Nasal carriage rate of *Staphylococcus aureus* in close contacts of AD patients	Observational study	3b
Graefe T [20]	2001	UVA1 treatment for skin nodular disease	Case report	4
Krutmann J [21]	1998	High‐dose UVA1 treatment for AD	Multicenter RCT	1b
Tzaneva S [22]	2001	Comparison of high‐dose vs. medium‐dose UVA1 treatment for severe AD	RCT	1b
Staab D [23]	2006	Age‐stratified structured education for managing children with AD	Multicenter RCT	1b
Ersser SJ [24]	2014	Psychological and educational interventions for children with AD	Cochrane systematic review	1a
Wilken B [25]	2023	Comprehensive review of education for AD patients	Scoping review	2a
Evers AW [26]	2009	Multidisciplinary training on itch sensitivity in adults with AD	RCT	1b
LeBovidge J [27]	2017	Education, shared decision‐making, and healthcare accessibility in AD treatment	Expert opinion/review	4
Singleton H [28]	2024	Effects of education and psychological interventions in AD	Cochrane systematic review	1a
Munday J [29]	2002	Efficacy of chlorpheniramine for itch in children with AD	RCT (negative result)	1b
van Zuuren EJ [30]	2014	Systematic review summary of oral H1 antihistamines as monotherapy for AD	Cochrane review summary	1a
van der Schaft J [31]	2015	Impact of long‐term cyclosporine A treatment on serum creatinine in AD patients	Cohort study	2b
Beck LA [32]	2014	Dupilumab treatment for adults with moderate to severe AD	RCT	1b
Blauvelt A [33]	2017	Long‐term management of dupilumab combined with topical steroids (LIBERTY AD CHRONOS)	Phase III RCT	1b
Simpson EL [34]	2016	Two Phase III trials of dupilumab for AD	Phase III RCT	1b
Ruzicka T [35]	2017	Anti‐IL‐31 receptor an antibody treatment for AD	RCT	1b
El‐Khalawany MA [36]	2013	Comparison of methotrexate and cyclosporine for severe pediatric AD	Multicenter RCT	1b
Schram ME [37]	2011	Comparison of methotrexate and azathioprine for severe AD	RCT	1b
Darsow U [38]	2011	Application of allergen‐specific immunotherapy (AIT) in AD	Review	4
Darsow U [39]	2012	Updates on AIT for AD treatment	Expert opinion/review	4
Welsh SE [40]	2021	Effects of Neurokinin‐1 receptor antagonist tradipitant on itch in atopic dermatitis	Multicenter RCT	1b
González‐López G [41]	2017	Efficacy and safety of wet wrap treatment for patients with atopic dermatitis: systematic review and meta‐analysis	Systematic review	2a

**TABLE 2:** Physician‐directed topical therapies for pruritus in atopic dermatitis.

Intervention	Evidence level	Recommendation strength	Itch outcome (effect size vs. control, 95% CI)	Subgroup findings	Clinical recommendation
Topical corticosteroids (TCS)	1a	A	NR (heterogeneous agents, potencies, and itch scales across trials; pruritus‐specific pooled effect sizes were not consistently reported in comparable metrics)	NR	Recommended as first‐line therapy for acute flares of AD‐associated pruritus. TCS can rapidly reduce inflammation and itch and remain the cornerstone of short‐term topical management. Potency and duration should be individualized according to disease severity, body site, and age
Topical calcineurin inhibitors (TCI; e.g., tacrolimus, pimecrolimus)	1a	A	NR (itch‐specific effect sizes with 95% CI were not consistently extractable in a unified metric across included studies)	Particularly useful for sensitive areas such as the face, neck, eyelids, and flexural sites	Recommended for sensitive skin areas and for longer‐term maintenance strategies, particularly when steroid‐sparing treatment is preferred. TCIs reduce recurrence risk and avoid steroid‐induced skin atrophy
Topical JAK inhibitors (e.g., ruxolitinib cream)	1b	A	Ruxolitinib 1.5% vs. vehicle at Week 8: between‐group difference in Itch NRS response = 36.8% (95% CI 25.7% to 47.9%)	Subgroup findings not consistently reported in accessible summary sources	Recommended for patients with mild‐to‐moderate or moderate AD requiring rapid itch reduction. Available evidence suggests strong antipruritic efficacy, although superiority over potent TCS or tacrolimus has not been consistently established across all analyses
Topical antibiotics/antimicrobial measures for infected lesions	2b	B	NR (itch outcomes were usually secondary endpoints; effect sizes with CI were not consistently reported)	Most relevant when secondary bacterial infection or *Staphylococcus aureus* colonization is present	Not recommended as routine antipruritic therapy, but may be considered when clinical infection or bacterial colonization contributes to disease worsening. Use should be guided by infection status rather than itch alone

### 3.2 Evidence Summary for Clinical Management of Pruritus in Atopic Dermatitis

#### 3.2.1 Physician‐Directed Therapies

The best‐available evidence for physician‐directed therapies is summarized in Tables 2 and 3. These therapies include topical anti‐inflammatory treatments, systemic therapies, biologics, JAK inhibitors, and phototherapy, all of which are primarily initiated and adjusted by physicians. Where available, we summarize representative effect sizes with corresponding 95% confidence intervals (95% CI) for pruritus‐related outcomes and highlight subgroup findings by disease severity, age group, or ethnicity.

**TABLE 3:** Physician‐directed systemic therapies for pruritus in atopic dermatitis.

Intervention	Evidence level	Recommendation strength	Itch outcome (effect size vs. control, 95% CI)	Subgroup findings	Clinical recommendation
First‐generation sedating antihistamines (e.g., hydroxyzine, chlorpheniramine)	1b	D	No clinically meaningful antipruritic effect vs. placebo in RCTs; 95% CI not consistently reported in included sources	Pediatric safety concerns; avoid routine use in children	Not recommended as routine antipruritic therapy in AD. May be considered only for short‐term night‐time use in selected patients with severe sleep disturbance related to itch
Second‐generation H1 antihistamines (e.g., cetirizine)	1a–1b	B	NR (itch relief in AD is inconsistent and often indirect; many studies focus on concomitant allergic symptoms)	More relevant in patients with allergic rhinitis, urticaria, or other histamine‐mediated comorbidities	Not recommended as monotherapy for AD‐related pruritus. May be considered when comorbid allergic disease is also present
Neuromodulators (e.g., tradipitant)	1b	B	NR (effect sizes with 95% CI not extractable in a consistent format from available sources)	NR	May provide benefit in selected patients, particularly when sleep disturbance and neurogenic itch features are prominent, but current evidence remains mixed
Cyclosporine A	1b	B	Standardized mean difference (SMD) in itch improvement: −1.28 (95% CI −2.30 to −0.27)	Generally used in refractory moderate‐to‐severe disease	Recommended for short‐term use in refractory moderate‐to‐severe AD with substantial pruritus burden. Requires careful monitoring for nephrotoxicity, hypertension, and relapse after discontinuation
Methotrexate (MTX)	2b	B	NR (itch‐specific effect sizes with 95% CI not consistently reported in accessible sources)	NR	May be considered as an alternative systemic immunosuppressant in refractory AD, particularly when cyclosporine is not suitable
Allergen‐specific immunotherapy (ASIT)	2a	B	NR (itch outcomes variably defined; effect sizes with 95% CI not consistently available)	Potentially more relevant in patients with confirmed aeroallergen sensitization	May be considered in selected sensitized patients, but evidence is indirect and applicability is limited to carefully phenotyped cases
Dupilumab (anti‐IL‐4/IL‐13)	1a	A	SMD in itch improvement: −0.43 (95% CI −0.59 to −0.28)	Benefits demonstrated across moderate‐to‐severe AD populations; limited subgroup‐specific reporting in included sources	Strongly recommended for moderate‐to‐severe AD with significant pruritus burden. Demonstrates meaningful improvement in itch, skin inflammation, and long‐term disease control
Nemolizumab (anti‐IL‐31 receptor)	1b	B	SMD in itch improvement: −0.46 (95% CI −0.77 to −0.15)	Rapid onset of antipruritic effect reported in trial populations	Recommended as a promising targeted option for patients with prominent itch, especially where IL‐31‐driven pruritus is a key clinical feature
Systemic JAK inhibitors (e.g., upadacitinib, abrocitinib, baricitinib)*	1b	A	NR in current summary table (rapid itch reduction consistently reported across phase 3 analyses, but effect measures were heterogeneous across trials)	Most evidence derived from moderate‐to‐severe AD populations	Strongly recommended for appropriately selected patients with moderate‐to‐severe AD requiring rapid itch reduction, while recognizing the need for careful risk assessment and ongoing safety monitoring
Narrowband UVB (NB‐UVB) phototherapy	1a	A	Mean itch reduction (VAS): −3.5 points (95% CI −4.2 to −2.8) vs. baseline	Comparable efficacy to cyclosporine in one RCT	Recommended as an effective physician‐directed treatment option for chronic AD‐associated pruritus, especially when systemic therapy is not preferred or not tolerated
Medium‐dose UVA1 phototherapy	1b	A	Itch reduction (SCORAD pruritus component): −2.1 points (95% CI −2.8 to −1.4) vs. baseline	Stronger effect than low‐dose UVA1 in severe AD	Recommended for acute severe AD with marked inflammation and itch when phototherapy is available and clinically appropriate

* *p* < 0.05.

#### 3.2.2 Nursing‐Implemented or Nursing‐Supported Interventions

The best‐available evidence for nursing‐implemented or nursing‐supported interventions is summarized in Table 4. These interventions include skin barrier care, bathing and wet‐wrap practices, trigger avoidance, patient education, behavioral support, adherence promotion, and follow‐up strategies. Evidence levels, recommendation strengths, and key outcomes are presented where available. Region‐specific or traditional therapies with limited international generalizability are presented separately in Table S3 to avoid overgeneralization of findings to broader clinical settings.

**TABLE 4:** Nursing‐implemented or nursing‐supported interventions for pruritus in atopic dermatitis.

Intervention	Evidence level	Recommendation strength	Recommendations	Key outcomes (effect size [95% CI])
Wet wrap therapy	1b	A	Recommended as a short‐term nursing‐supported intervention for acute exudative or severe flares when prescribed, particularly in combination with diluted topical corticosteroids and careful monitoring of tolerability	Mean pruritus NRS reduction: −2.8 [95% CI −3.3 to −2.3] vs. TCS alone; improved TIS scores in children
Emollients	2a	B	Core nursing‐supported intervention for all patients with AD; regular and sufficient use of emollients is recommended to improve skin barrier function, reduce xerosis, and alleviate itch. Emollients containing urea or glycerin may provide additional benefit	Meta‐analysis: RR of itch improvement with urea‐based emollient vs. vehicle: 1.45 [95% CI 1.10–1.91]; mean TEWL reduction: −3.2 g/m^2^/h
Patient education programs	1b	A	Strongly recommended as a nursing‐supported intervention to improve self‐management, adherence, trigger avoidance, and long‐term symptom control; structured education programs were associated with clinically meaningful improvements in itch and quality of life	Mean pruritus reduction (POEM score): −3.6 [95% CI −5.1 to −2.1]; improved QoL (DLQI): −4.0 points [95% CI −5.5 to −2.5] vs. usual care
Psychological and behavioral interventions	1a	A	Recommended, particularly in patients with stress‐related scratching, sleep disturbance, or maladaptive itch behaviors; psychological counseling and CBT can help reduce scratching and improve the itch‐scratch cycle	Cognitive‐behavioral therapy: pruritus reduction NRS −2.4 [95% CI −3.2 to −1.6]; improved sleep quality and DLQI in children and adolescents
Bathing and daily skin care guidance	4–5	B	Recommend lukewarm bathing, limited bathing duration, prompt post‐bath emollient application, and avoidance of harsh cleansers or overheating; these measures support barrier repair and reduce itch exacerbation	No pooled comparative effect size available; supported by guideline and expert‐consensus recommendations for barrier‐oriented AD care
Adherence Support and Follow‐up	4–5	B	Regular follow‐up, reinforcement of treatment technique, assessment of steroid phobia, and review of barriers to adherence are recommended to improve long‐term itch control and treatment effectiveness	Quantitative pooled effect sizes not consistently reported; supported by education, behavioral, and nursing‐practice literature

### 3.3 Nursing‐Specific Roles in Itch Management of Atopic Dermatitis

Based on the evidence summarized above, nursing‐specific roles in itch management were identified and are presented in Table 5, focusing on pruritus and symptom assessment, skin barrier care and topical treatment guidance, patient education and behavioral management, subcutaneous biologic therapy support, safety monitoring, adherence support, follow‐up, and criteria for referral or treatment escalation.

**TABLE 5:** Nursing‐specific roles in itch management of atopic dermatitis: key practice recommendations.

Nursing domain	Recommendations	Practical tips	Supporting evidence
Pruritus and symptom assessment	Use validated tools such as the Visual Analog Scale (VAS), Numerical Rating Scale (NRS), or pruritus‐related items within SCORAD/POEM to assess itch intensity, nocturnal worsening, sleep disturbance, scratching behavior, and quality‐of‐life impact. These tools are simple, reliable, and useful for baseline assessment and follow‐up monitoring of treatment response	Document baseline itch severity and reassess at follow‐up Ask specifically about night‐time worsening, sleep loss, and scratching patterns Record symptom trends alongside treatment changes	Supported by trials and reviews emphasizing standardized symptom assessment and itch‐related outcomes in AD
Skin barrier care and topical treatment guidance	Provide structured guidance on moisturization frequency, bathing practices, wet‐wrap use when indicated, and correct application of topical anti‐inflammatory agents such as topical corticosteroids, topical calcineurin inhibitors, PDE4 inhibitors, or topical JAK inhibitors. Nurses should also address steroid phobia, reinforce fingertip‐unit instructions, and encourage consistent use of emollients and prescribed topical therapies	Encourage liberal emollient use, especially after bathing Teach fingertip‐unit dosing and stepwise topical application Use wet wraps short‐term when prescribed and indicated	Supported by evidence on emollients, wet‐wrap therapy, and topical anti‐inflammatory treatments
Patient education and behavioral management	Educate patients and families on common itch triggers and skin irritants, including dust mites, harsh cleansers, excessive heat, sweating, and scratching behaviors. Education should also include nail care, sleep hygiene, avoidance of long hot showers, trigger reduction, and practical strategies to interrupt the itch‐scratch cycle	Keep nails short and consider cotton gloves at night when appropriate Reinforce sleep routines and trigger avoidance plans Provide written instructions for home care and flare management	Supported by structured education programs, psychological interventions, and behavioral‐support evidence
Environmental and daily skin care guidance	Advise patients to maintain a comfortable indoor humidity level, avoid excessive heat exposure, use lukewarm rather than hot water for bathing, and apply moisturizers promptly after bathing. These measures help reduce xerosis, maintain barrier function, and minimize itch exacerbation	Recommend fragrance‐free cleansers and moisturizers Emphasize prompt post‐bath emollient use Review daily routines that may worsen dryness or overheating	Consistent with skin‐barrier care principles and non‐pharmacological management evidence
Infection control and skin care	Promptly identify signs of secondary infection and notify the treating physician when bacterial colonization or recurrent infections are suspected. Nurses should reinforce hygiene education, support implementation of prescribed antimicrobial measures, and monitor response to treatment	Watch for oozing, crusting, pustules, worsening erythema, or pain Reinforce gentle cleansing and hand hygiene Document response to prescribed antimicrobial measures	Supported by studies linking *Staphylococcus aureus* control with improvement in AD severity
Topical corticosteroids	Educate patients on appropriate potency selection according to physician prescription, fingertip‐unit application, treatment duration, and site‐specific precautions. Reinforce correct use on affected areas only, avoid overuse, and monitor for local adverse effects such as skin atrophy, irritation, or delayed barrier recovery	Clarify where, how often, and how long TCS should be used Highlight extra caution for face, groin, and flexural areas Check adherence and technique at each review	Supported by evidence and guideline recommendations identifying TCS as a core therapy for AD flares
Subcutaneous biologic therapy	Educate patients receiving subcutaneous biologic therapy on injection technique, storage requirements, site rotation, adherence to prescribed dosing schedules, and recognition of common adverse effects such as injection‐site reactions, ocular symptoms, hypersensitivity reactions, or other therapy‐specific adverse events. Arrange regular follow‐up to evaluate itch response, sleep improvement, treatment adherence, and tolerability, and promptly communicate persistent, worsening, or unexpected adverse events to the treating physician	Confirm patient confidence with self‐injection technique, device handling, storage, and safe disposal before home use Reinforce site rotation, regular dosing schedules, missed‐dose instructions, and maintenance therapy expectations Ask about injection‐site reactions, ocular symptoms, hypersensitivity symptoms, and other biologic‐specific adverse effects Reinforce scheduled follow‐up, adherence, and timely reporting of persistent or worsening symptoms	Supported by randomized trials and guideline recommendations showing that subcutaneous biologic therapies, including dupilumab and other targeted biologics, can improve itch, inflammation, sleep‐related outcomes, and long‐term disease control in appropriately selected patients with moderate‐to‐severe AD
Safety monitoring and follow‐up	Monitor treatment‐related adverse effects associated with topical and systemic therapies, assess adherence, and document changes in itch intensity, sleep, scratching behavior, and quality of life at each follow‐up. Nurses should identify warning signs requiring physician review, including treatment failure, worsening skin lesions, recurrent infection, intolerable adverse effects, or progressive sleep disturbance	Use a brief follow‐up checklist covering efficacy, tolerability, and adherence Escalate promptly when symptoms worsen or adverse effects persist Maintain clear documentation across visits	Supported by evidence summaries highlighting safety monitoring, adherence assessment, and follow‐up coordination
Stratified nursing process and referral criteria	For mild itch, prioritize skin barrier support, trigger avoidance, and adherence to prescribed topical therapy. For moderate itch with sleep disturbance or recurrent scratching, intensify assessment, education, behavioral support, and follow‐up. For severe or refractory itch, or when systemic treatment may be required, nurses should promptly coordinate referral or escalation to physician‐led management	Match nursing intensity to symptom severity and sleep impact Reassess every 2–4 weeks or sooner during flares Trigger referral when control remains suboptimal	Supported by the integrated evidence pathway for stepwise AD itch management

### 3.4 Proposed Nursing Care Process for Pruritus Management

Based on the reviewed evidence, a structured and stratified nursing care process can be proposed to support pruritus management, alleviate discomfort, and improve patient outcomes. The process begins with comprehensive assessment, in which itch intensity, nocturnal worsening, sleep disturbance, scratching behavior, skin barrier status, and quality‐of‐life impact are evaluated using standardized tools (e.g., NRS, VAS, SCORAD, or POEM) and documented at baseline and follow‐up visits. Based on the assessment findings, nurses implement a stepwise and severity‐based protocol. In patients with mild itch, priority is given to skin barrier optimization, moisturization, trigger avoidance, and reinforcement of prescribed topical therapy. In patients with moderate itch, especially when sleep disturbance or persistent scratching is present, nurses intensify symptom monitoring, patient education, behavioral strategies, and follow‐up frequency. In patients with severe, persistent, or treatment‐refractory itch, nurses coordinate timely escalation to physician‐led evaluation for systemic or advanced therapies while continuing supportive skin care, adherence counseling, and safety monitoring.

**FIGURE 2:**
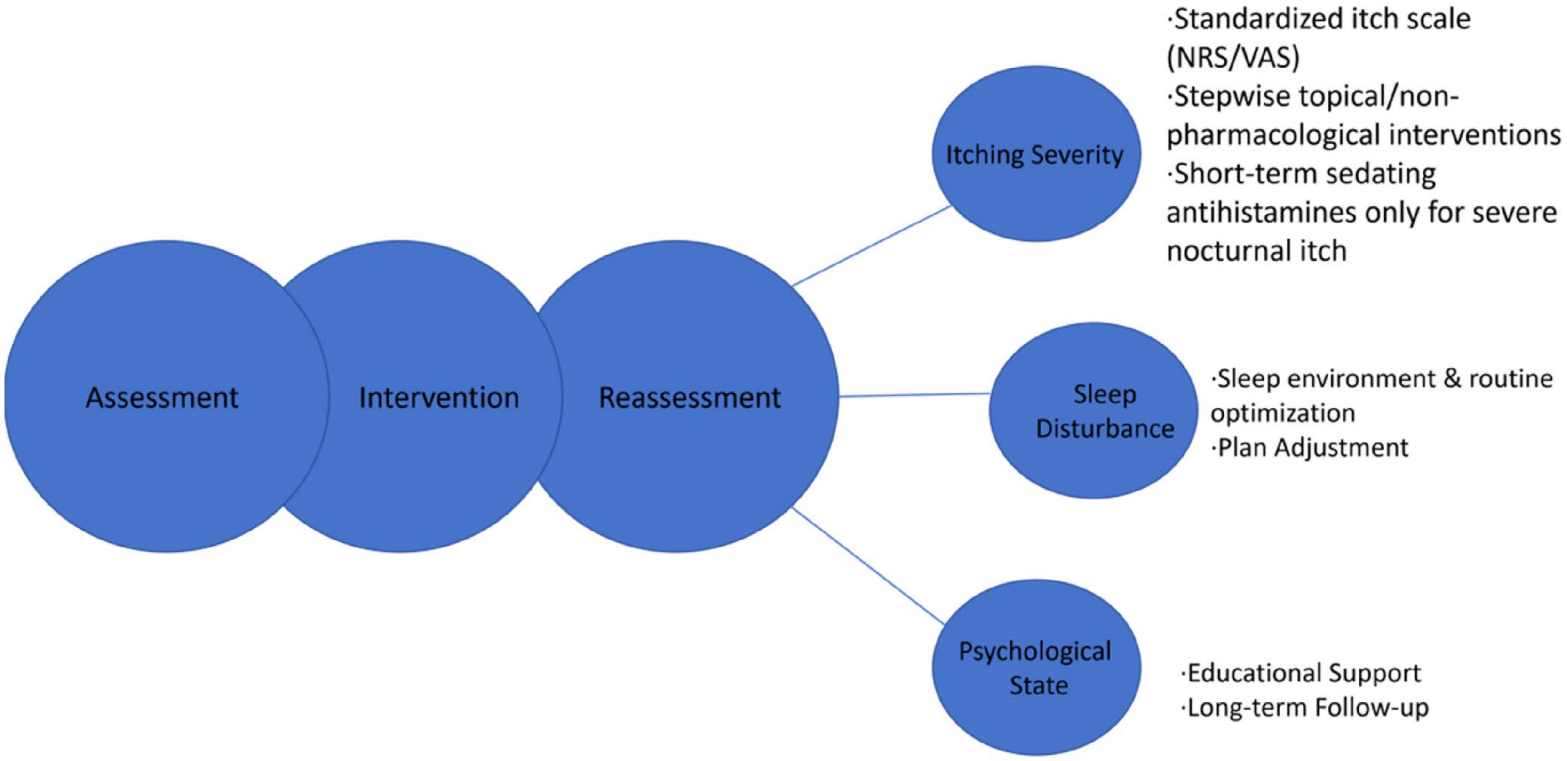
Stratified Nursing Care Pathway for Itch Management in Atopic Dermatitis. The pathway includes baseline assessment of itch intensity, nocturnal symptoms, sleep disturbance, scratching behavior, and quality‐of‐life impact; stratified nursing interventions for mild, moderate, and severe itch; reassessment every 2–4 weeks; and referral/escalation to physician‐led therapy when symptom control remains suboptimal or adverse events occur.

Sedating first‐generation antihistamines are not used routinely, but may be considered for short‐term, night‐time use in selected patients with severe nocturnal itch and sleep disturbance despite optimized topical therapy, while second‐generation antihistamines are reserved for those with concomitant allergic rhinitis or urticaria. Additionally, sleep disturbances caused by itching are addressed through tailored nursing measures, such as optimizing sleep environments, establishing regular sleep routines, and providing patient and family education. Finally, a re‐evaluation phase—typically every 2–4 weeks—ensures the effectiveness of the interventions by reviewing itch scores, sleep quality, and adherence; if symptom control remains suboptimal, nurses coordinate escalation to physician‐led systemic therapies in accordance with the evidence‐based pathway, as illustrated in Figure 2.

## 4 Discussion

### 4.1 Pharmacological Treatments

Topical corticosteroids, calcineurin inhibitors, and immunosuppressants such as cyclosporine are established first‐line therapies for atopic dermatitis. These agents, including corticosteroids, tacrolimus, and cyclosporine, are widely utilized due to their potent anti‐inflammatory properties and effectiveness in alleviating pruritus. Clinical trials indicate that long‐term use of corticosteroids is associated with a reduction in pruritus severity, achieved through the attenuation of inflammation, correction of immune dysregulation, and restoration of skin barrier function [42, 43]. Tacrolimus and pimecrolimus, commonly used calcineurin inhibitors, inhibit T lymphocyte‐mediated inflammatory responses and suppress the synthesis of pro‐inflammatory cytokines. Research shows that pimecrolimus significantly improves pruritus compared to its vehicle; however, betamethasone valerate demonstrates superior efficacy in reducing pruritus across all concentrations. Cyclosporine is an immunosuppressant that mainly targets T lymphocyte activation and proliferation. By doing so, it decreases the release of inflammatory mediators, helping to alleviate skin inflammation and itching. This medication is especially advantageous for patients suffering from severe, refractory atopic dermatitis who have not found relief with standard treatments. It can provide quick relief from distressing symptoms; however, its effects are typically short‐lived, and many patients experience a high rate of relapse over time [44]. When comparing tacrolimus and cyclosporine, tacrolimus demonstrates a faster onset of action, with both medications exhibiting comparable safety profiles. These findings support the preferential use of topical 0.1% tacrolimus in the management of atopic dermatitis due to the potential adverse effects associated with cyclosporine. Overall, pharmacological decision‐making in moderate‐to‐severe AD remains primarily physician‐directed, including the initiation, adjustment, and discontinuation of topical, systemic, biologic, and phototherapy‐based treatments. In contrast, the core contribution of nursing practice lies in structured symptom assessment, patient and family education, skin care support, treatment implementation, safety monitoring, identification of adherence barriers, and long‐term follow‐up coordination. Clarifying this boundary is important to avoid overstating the role of nursing in therapeutic prescribing while highlighting its essential role in optimizing treatment delivery, continuity of care, and patient‐centered outcomes.

Dupilumab, an IL‐4/IL‐13 inhibitor, diminishes inflammation and pruritus by blocking the signaling pathways of these interleukins. A meta‐analysis conducted by Silverberg et al. [45], based on four Phase 3 randomized trials (SOLO 1 [34], SOLO 2 [34], AD ADOL [46], and CHRONOS [33]), demonstrated that dupilumab treatment leads to rapid and sustained improvements in pruritus severity from the first dose, with responses progressively increasing and persisting throughout the treatment period, lasting up to 1 year. Janus kinase (JAK) inhibitors such as upadacitinib, baricitinib, and tralokinumab inhibit the JAK pathway, thereby mitigating the transmission of inflammatory and pruritic signals. A post hoc analysis of three Phase 3 studies (Heads Up and MeasureUp 1/2) compared the efficacy of upadacitinib against dupilumab or placebo in moderate to severe atopic dermatitis patients, revealing that upadacitinib 30 mg was superior to dupilumab in achieving skin clearance and reducing pruritus, with both upadacitinib 15 and 30 mg demonstrating advantages over placebo. These benefits may translate into greater improvements in patients' quality of life [47]. Upadacitinib combined with topical corticosteroids provides rapid and sustained enhancements in atopic dermatitis symptoms and overall quality of life [48]. Post hoc analyses from three Phase 3 studies (BREEZE‐AD1, BREEZE‐AD2, BREEZE‐AD7) indicated that baricitinib effectively alleviates pruritus and sleep disturbances in atopic dermatitis patients, typically within one day following the initial dose. In studies where baricitinib was used in conjunction with topical corticosteroids, improvements in pruritus and sleep disturbances correlated with enhanced quality of life, productivity, and overall therapeutic benefit. Nemolizumab, targeting the IL‐31 receptor alpha subunit, modulates neuroimmune responses by directly blocking signaling pathways, thereby alleviating pruritus. Existing literature suggests its efficacy in reducing pruritus and improving symptoms in children aged 6–12 with atopic dermatitis. Abrocitinib, an oral JAK1 inhibitor, demonstrated significant pruritus relief as early as Day 4 of treatment in subgroup analyses of the Phase III JADE COMPARE trial, with pruritus relief by Week 2 correlating with skin clearance and improved quality of life by Week 12. Multiple clinical trial analyses indicate its substantial effectiveness in moderate to severe pruritic atopic dermatitis, providing rapid, profound, and sustained improvements in both pruritus and quality of life. Lebrikizumab, which targets IL‐13, showed sustained improvements in pruritus and sleep disturbances from Week 16 to Week 52 in follow‐up analyses from the ADvocate1 and ADvocate2 studies. However, all currently approved systemic JAK inhibitors carry boxed warnings for serious infections, malignancy, major adverse cardiovascular events and thrombosis. From a nursing perspective, initiation and follow‐up of systemic JAK inhibitor therapy should include baseline assessment of cardiovascular and thrombotic risk factors, history of malignancy and chronic infection, and laboratory evaluation of complete blood count, liver enzymes, renal function and lipid profile, together with screening for latent tuberculosis and viral hepatitis according to local guidelines. During treatment, nurses should monitor for laboratory abnormalities and clinical signs of infection, thrombosis or cardiovascular events and ensure timely communication with physicians. Patient counseling should emphasize infection‐prevention measures, prompt reporting of fever, cough, dyspnea or unilateral leg swelling, and vaccination planning—ideally updating inactivated vaccines before starting therapy and avoiding live vaccines while receiving systemic JAK inhibitors.

Histamine, a well‐known pruritogen, has led to the widespread use of H1 antihistamines for managing pruritus in atopic dermatitis; however, their efficacy in addressing refractory pruritus remains limited [49]. Second‐generation antihistamines offer reduced sedation compared to first‐generation agents and exhibit additional efficacy in blocking various mediators. This suggests that second‐generation H1 antihistamines may present advantages regarding safety, particularly in patients with concomitant allergic rhinitis or urticaria, but they should not be considered first‐line antipruritic agents in atopic dermatitis [43]. Some studies indicate that H1 and H2 antihistamines do not significantly alleviate pruritus or inflammation in AD patients. Additionally, a randomized clinical investigation conducted across multiple centers examined the efficacy of the H1‐receptor antagonist chlorpheniramine versus placebo in 155 pediatric patients with atopic dermatitis. The double‐masked study demonstrated comparable outcomes between active treatment and control groups for nocturnal pruritus and associated scratching behaviors [29]. Taken together, these findings support a restricted role for H1 antihistamines in AD, with first‐generation agents used, if at all, only for their short‐term sedative effects in severe nocturnal itch, and second‐generation agents reserved for patients with comorbid allergic symptoms rather than as routine antipruritic monotherapy.

Traditionally, therapeutic approaches for AD‐associated pruritus have primarily focused on two key pathophysiological mechanisms: epidermal barrier repair and inflammatory cascade modulation. However, there is a growing emphasis on directly targeting the neural pathways mediating pruritus signals. Neuromodulatory agents may aid in alleviating pruritus in atopic dermatitis patients, with centrally acting antipruritic and anti‐neurogenic agents representing promising therapeutic avenues [50, 51]. Additionally, research indicates that Tradipitant (a neurokinin‐1 receptor antagonist) exhibits mixed effects on pruritus in atopic dermatitis [40]. Certain pharmacological and traditional interventions summarized in this review—such as compound glycyrrhizin, Tripterygium wilfordii polyglycosides, total glycosides of paeony, and traditional Chinese medicine baths or topical preparations—primarily reflect regional practice patterns in East Asia and may not be universally available, regulated, or guideline‐endorsed across countries. Although some of these agents were supported by trial‐level evidence, the recommendation strength for compound glycyrrhizin and Tripterygium wilfordii polyglycosides was downgraded to Grade C because of safety concerns, limited international generalizability, and the need for closer physician‐directed monitoring than many other available therapies. Their evidence base, accessibility, and acceptability differ substantially between healthcare systems. These interventions were therefore presented separately in Table S3 to acknowledge their regional relevance while avoiding overgeneralization to an international audience. Interpretation of these findings should take local regulatory approval, resource availability, regional guideline context, and safety‐monitoring requirements into account. In particular, compound glycyrrhizin requires attention to blood pressure, serum electrolytes, fluid retention, and related adverse effects, while Tripterygium wilfordii preparations require careful monitoring for systemic toxicity. Therefore, these therapies should be regarded as optional, region‐specific adjuncts rather than broadly recommended treatments. Across the pharmacological trials included, most enrolled patients with moderate‐to‐severe disease, and only a minority reported stratified outcomes by baseline severity, age or ethnicity; where such subgroup data were available, they did not reveal large qualitative differences in treatment effects, but incomplete and heterogeneous reporting precluded formal quantitative subgroup analyses. Overall, our pharmacological recommendations are broadly consistent with contemporary international and national atopic dermatitis guidelines published between 2020 and 2024, which emphasize individualized selection of topical, systemic and biologic therapies according to disease severity, comorbidities and patient preferences.

### 4.2 Summary of Evidence for Non‐Pharmacological Treatments

Family constellation workshops constitute a psychological intervention model that enables patients to better understand familial dynamics, address familial secrets, and enhance interactions with their social environment, consequently alleviating stress. Research indicates that this approach can reduce pruritus severity and improve sleep quality and overall functioning in patients with atopic dermatitis and psoriasis [52]. Cross‐sectional survey studies have identified negative correlations between mindfulness aspects—such as “mindful action,”“acceptance and non‐judgmental orientation,” and “non‐reactive orientation”—and catastrophizing pruritus in atopic dermatitis patients. This suggests that psychological interventions emphasizing these mindfulness components may help mitigate patients' catastrophizing cognitive patterns regarding pruritus, thereby reducing its impact [53].

Comparative studies on the efficacy of narrowband ultraviolet B (NB‐UVB) radiation versus cyclosporine in treating pruritus associated with atopic dermatitis have demonstrated the effectiveness of NB‐UVB in alleviating pruritus [54]. Moreover, NB‐UVB has demonstrated effectiveness that is at least on par with broadband UVB in the treatment of chronic itching [55]. Additionally, both UVA/NBUVB and NBUVB combination phototherapy show similar clinical efficacy and safety in managing chronic atopic dermatitis [56]. Research on short‐term thermal application therapy in atopic dermatitis patients revealed that a 5‐s application at 49°C significantly and immediately alleviated pruritus, with effects enduring for an extended period and no habituation effects observed upon repeated applications. This indicates its potential as a non‐pharmacological, personalized approach to managing pruritus in atopic dermatitis, albeit with notable individual variability [57]. Across the included pharmacological and non‐pharmacological trials, only a minority explicitly reported minimal clinically important differences (MCIDs) for itch or quality‐of‐life scales, and quality‐of‐life outcomes such as sleep disturbance and daily functioning were often underreported, which limits the precision with which we can judge the clinical relevance of some observed effect sizes. From a nursing perspective, long‐term adherence to emollient use, topical anti‐inflammatory regimens, scheduled phototherapy, and, where indicated, systemic therapies is a key determinant of sustained itch control. Common barriers include complex treatment regimens, “steroid phobia,” concerns about adverse effects, and treatment fatigue; structured patient education, shared decision‐making, and regular follow‐up are therefore essential strategies to support adherence and optimize real‐world effectiveness. These findings further emphasize that the principal value of nursing in AD pruritus management lies not in prescribing treatment, but in assessment, education, monitoring, adherence support, and continuity of care across the disease course. In addition, the applicability of some interventions may vary across countries because of differences in healthcare delivery models, resource availability, reimbursement structures, cultural practices, and guideline recommendations; therefore, international interpretation of the present findings should be undertaken with attention to local practice context.

### 4.3 Nursing Boundaries and Practice Implications

Most pharmacological therapies discussed in this review are initiated and adjusted by physicians, particularly biologics, systemic immunomodulators, JAK inhibitors, and phototherapy‐based treatment strategies. For subcutaneous biologic therapies, nursing practice should emphasize universal treatment‐support tasks, including patient education on storage, injection technique, site rotation, device handling, safe disposal, maintenance scheduling, adherence, and recognition of injection‐site reactions or other therapy‐specific adverse effects. Nursing practice should therefore not be framed as directing therapeutic prescribing, but rather as providing essential support through symptom assessment, patient education, treatment monitoring, adherence promotion, and long‐term follow‐up. This distinction helps define the unique and clinically meaningful role of nursing within multidisciplinary AD care.

### 4.4 International Applicability and Generalizability

The international applicability of the evidence summarized in this review should be interpreted cautiously. Although many interventions, such as topical anti‐inflammatory therapies, biologics, JAK inhibitors, emollients, education programs, and wet‐wrap therapy, are broadly relevant across settings, some interventions are influenced by regional treatment traditions, local regulatory environments, and healthcare system differences. In particular, region‐specific or traditional therapies may have limited generalizability beyond the settings in which they were studied. Therefore, implementation of the present findings should be considered in conjunction with local clinical guidelines, treatment availability, and cultural context.

## 5 Conclusion

Effective pruritus management in atopic dermatitis requires a comprehensive, evidence‐based approach that integrates pharmacological and non‐pharmacological interventions while considering individual patient characteristics and long‐term adherence. This review summarizes current evidence and highlights key implications for nursing practice, particularly in symptom assessment, skin barrier care, patient education, adherence support, safety monitoring, and follow‐up. Through multidisciplinary collaboration, nursing practice can make an important contribution to improving pruritus control and advancing patient‐centered care.

## Author Contributions

Yuzhen She is responsible for the guarantor of integrity of the entire study, study concepts and design, literature research, data acquisition and analysis, manuscript preparation and editing and review; Qian Shen is responsible for the guarantor of integrity of the entire study, literature research, definition of intellectual content, data analysis, statistical analysis; Manni Chen is responsible for the data analysis, literature research, manuscript preparation and editing; Rufang Liu is responsible for the guarantor of integrity of the entire study, literature research, statistical analysis, study concepts, definition of intellectual content. All authors read and approved the final manuscript.

## Funding

This study is funded by the Special Fund for Research on Traditional Chinese Medicine Science and Technology in Guangdong Provincial Hospital of Traditional Chinese Medicine, No. YN2022HL17 (to Shen Qian).

## Ethics Statement

All analyses were based on previously published studies; thus, no ethical approval and patient consent are required.

## Consent

The authors have nothing to report.

## Conflicts of Interest

The authors declare no conflicts of interest.

## Supporting information


**Table S1:** Overview of risk‐of‐bias assessments for randomized controlled trials and cohort studies.
**Table S2:** Definitions of Level of Evidence and Strength of Recommendation.
**Table S3:** Region‐Specific or Traditional Therapies for Pruritus in Atopic Dermatitis.

## Data Availability

The datasets used or analyzed during the current study are available from the responding author on reasonable request.
