# Supplementary material for: Evidence Summary of Pruritus Management in Patients With Atopic Dermatitis: Implications for Nursing Practice
Source: J Cosmet Dermatol. 2026 Aug 31;25(9):e71111. doi: 10.1111/jocd.71111 (PMC13530417; doi:10.1111/jocd.71111)
Supplement: Supplementary file 1 [file JOCD-25-e71111-s001.docx]

Supplementary Table 1. Overview of risk-of-bias assessments for randomized controlled trials and cohort studies

| **First author (year)** | **Study type** | **Random sequence generation** | **Allocation concealment** | **Blinding of participants and personnel** | **Blinding of outcome assessment** | **Incomplete outcome data** | **Selective reporting** | **Other bias** | **Overall risk of bias** |
| --- | --- | --- | --- | --- | --- | --- | --- | --- | --- |
| Sigurgeirsson B (2015) | Randomized controlled trial | Low | Low | Low | Low | Some concerns | Some concerns | Some concerns | Some concerns |
| Silverberg JI (2021) | Phase III double-blind RCT | Low | Low | Low | Low | Some concerns | Some concerns | Some concerns | Some concerns |
| Huang JT (2009) | Cohort study | Not applicable | Not applicable | High | Some concerns | Some concerns | Some concerns | High (residual confounding) | High |
| Krutmann J (1998) | Multicenter RCT | Some concerns | Some concerns | High | Some concerns | Some concerns | Some concerns | Some concerns | High |
| Tzaneva S (2001) | RCT | Some concerns | Some concerns | High | Some concerns | Some concerns | Some concerns | Some concerns | High |
| Staab D (2006) | Multicenter RCT (educational intervention) | Some concerns | Some concerns | High | Some concerns | Some concerns | Some concerns | Some concerns | High |
| Evers AW (2009) | RCT (multidisciplinary training) | Some concerns | Some concerns | High | Some concerns | Some concerns | Some concerns | Some concerns | High |
| Munday J (2002) | RCT | Some concerns | Some concerns | Low | Low | Some concerns | Some concerns | High (small sample size, short follow-up) | High |
| van der Schaft J (2015) | Cohort study | Not applicable | Not applicable | High | Some concerns | Some concerns | Some concerns | High (confounding, observational design) | High |
| Beck LA (2014) | Phase II/III RCT | Low | Low | Low | Low | Some concerns | Some concerns | Some concerns | Some concerns |
| Blauvelt A (2017) | Phase III RCT | Low | Low | Low | Low | Some concerns | Some concerns | Some concerns | Some concerns |
| Simpson EL (2016) | Phase III RCT | Low | Low | Low | Low | Some concerns | Some concerns | Some concerns | Some concerns |
| Ruzicka T (2017) | RCT | Low | Low | Low | Low | Some concerns | Some concerns | Some concerns | Some concerns |
| El-Khalawany MA (2013) | Multicenter RCT | Some concerns | Some concerns | High | Some concerns | Some concerns | Some concerns | Some concerns | High |
| Schram ME (2011) | RCT | Some concerns | Some concerns | High | Some concerns | Some concerns | Some concerns | Some concerns | High |
| Welsh SE (2021) | Multicenter RCT | Low | Low | Low | Low | Some concerns | Some concerns | Some concerns | Some concerns |

Supplementary Table 2. Definitions of Level of Evidence and Strength of Recommendation

| **Level of Evidence** | **Definition** |
| --- | --- |
| 1a | Meta-analysis or high-quality systematic review of RCTs |
| 1b | Individual RCT |
| 2a | Systematic review of cohort studies |
| 2b | Individual cohort study |
| 3b | Case-control or observational study |
| 4 | Case series or expert opinion |
| 5 | Guideline, consensus, or position statement |

Supplementary Table 3. Region-Specific or Traditional Therapies for Pruritus in Atopic Dermatitis

| **Intervention** | **Evidence Level** | **Recommendation Strength** | **Key Evidence Summary** | **Clinical Considerations / Limitations** |
| --- | --- | --- | --- | --- |
| Compound glycyrrhizin | 1b | C | Reported to have anti-inflammatory and immunomodulatory effects and has been used in some regional clinical settings for AD symptom control, including pruritus. | Primarily reflects regional practice patterns; international availability and guideline endorsement are limited. Given the potential for blood pressure elevation, electrolyte disturbances, fluid retention, edema, and weight gain, compound glycyrrhizin should be used cautiously and requires close clinical monitoring and physician-directed management. |
| Tripterygium wilfordii polyglycosides | 1b | C | Reported to exert anti-inflammatory and immunosuppressive effects and has been used in some studies for AD management. | Primarily used in East Asian settings; broader generalizability is limited. Because of potential serious systemic adverse effects, this therapy should be used cautiously only in selected patients and requires close physician-directed monitoring of blood count, urinalysis, liver and kidney function, and ECG. |
| Total glycosides of paeony | 1b | B | Reported to have anti-inflammatory and immunomodulatory properties and has been used as an adjunctive option in some regional AD treatment settings. | International applicability is limited. Gastrointestinal adverse effects such as loose stools or diarrhea may occur. |
| Traditional XXX medicine washing / medicinal bath | 2b | B | May relieve pruritus and improve skin condition as adjunctive therapy in some studies, but evidence is derived mainly from small and heterogeneous studies. | Region-specific intervention with limited external validity for international audiences. Should not be considered a routine first-line intervention and should only be interpreted as adjunctive to standard care. |
| Traditional XXX medicine topical cream | 2b | B | Has been used mainly in children with dry, scaling, or lichenified lesions and may support skin hydration and barrier function in some small studies. | Evidence is limited and heterogeneous, with uncertain comparability to standard topical therapy. Should be considered adjunctive rather than a substitute for guideline-recommended treatment. |
